# Impact of preservation method and storage period on ribosomal metabarcoding of marine microbes: Implications for remote automated samplings

**DOI:** 10.3389/fmicb.2022.999925

**Published:** 2022-09-07

**Authors:** Matthias Wietz, Katja Metfies, Christina Bienhold, Christian Wolf, Felix Janssen, Ian Salter, Antje Boetius

**Affiliations:** ^1^Deep-Sea Ecology and Technology, Alfred Wegener Institute Helmholtz Centre for Polar and Marine Research, Bremerhaven, Germany; ^2^Max Planck Institute for Marine Microbiology, Bremen, Germany; ^3^Polar Biological Oceanography, Alfred Wegener Institute Helmholtz Centre for Polar and Marine Research, Bremerhaven, Germany; ^4^Helmholtz Institute for Functional Marine Biodiversity at the University of Oldenburg, Oldenburg, Germany; ^5^Faroe Marine Research Institute, Torshavn, Faroe Islands; ^6^MARUM Center for Marine Environmental Sciences, University of Bremen, Bremen, Germany

**Keywords:** seawater microbiome, sample preservation, DNA extraction, amplicon sequencing, 16S rRNA, 18S rRNA, autonomous sampling, time-series

## Abstract

Automated sampling technologies can enhance the temporal and spatial resolution of marine microbial observations, particularly in remote and inaccessible areas. A critical aspect of automated microbiome sampling is the preservation of nucleic acids over long-term autosampler deployments. Understanding the impact of preservation method on microbial metabarcoding is essential for implementing genomic observatories into existing infrastructure, and for establishing best practices for the regional and global synthesis of data. The present study evaluates the effect of two preservatives commonly used in autosampler deployments (mercuric chloride and formalin) and two extraction kits (PowerWater and NucleoSpin) on amplicon sequencing of 16S and 18S rRNA gene over 50 weeks of sample storage. Our results suggest the combination of mercuric chloride preservation and PowerWater extraction as most adequate for 16S and 18S rRNA gene amplicon-sequencing from the same seawater sample. This approach provides consistent information on species richness, diversity and community composition in comparison to control samples (nonfixed, filtered and frozen) when stored up to 50 weeks at *in situ* temperature. Preservation affects the recovery of certain taxa, with specific OTUs becoming overrepresented (SAR11 and diatoms) or underrepresented (*Colwellia* and pico-eukaryotes) after preservation. In case eukaryotic sequence information is the sole target, formalin preservation and NucleoSpin extraction performed best. Our study contributes to the design of long-term autonomous microbial observations in remote ocean areas, allowing cross-comparison of microbiome dynamics across sampling devices (e.g., water and particle samplers) and marine realms.

## Introduction

Microbial communities have fundamental ecological and biogeochemical roles in nutrient recycling and carbon sequestration (Jørgensen and Boetius, 2007; Fuhrman et al., 2015). Understanding the consequences of global change for marine ecosystems requires a robust assessment of microbial community dynamics over temporal and spatial scales (Sunagawa et al., 2015; Buttigieg et al., 2018). Automated sampling devices attached to observational platforms, e.g., ocean moorings, enable time-series observations of microbial dynamics (Herfort et al., 2016; Zhang et al., 2019, 2021). Typically combined with physical and chemical sensors, automated samplers are of particular value in remote and inaccessible areas, such as seasonally ice-covered environments (Liu et al., 2020; von Appen et al., 2021; Wietz et al., 2021; Ramondenc et al., 2022). For instance, autonomous sediment traps allow linking particle flux with microbial diversity over extended periods, based on microscopic counts (Salter et al., 2007, 2012, 2014; Nöthig et al., 2020; Zúñiga et al., 2021) and DNA sequencing (Metfies et al., 2017; Bachy et al., 2022; Valencia et al., 2022).

There is a growing toolbox and increasing application of automated water and particle sampling approaches (Supplementary Table S1). As *in situ* molecular analysis is still an emerging technology (Moore et al., 2021) and beyond the resource capacity of many observing programs, automated samplers mostly perform *in situ* preservation of sample material (Yamahara et al., 2019; Lindsay, 2021; Truelove et al., 2022). *In situ* preservation intends to minimize signal modification over the extended duration of device deployment and laboratory processing. Formalin and mercuric chloride are commonly used to preserve sinking particles in long-term monitoring programs (Lee et al., 1992; UNESCO-IOC, 1994; Bauerfeind et al., 2009; Lampitt et al., 2010; Fischer et al., 2016). Although these chemicals originally aimed to preserve tissues, particles and cells for bulk biogeochemical analyses, recent studies have demonstrated that downstream molecular analyses are feasible with both mercuric chloride- (Metfies et al., 2017) and formalin-fixed (Boeuf et al., 2019) sediment trap samples. Likewise, preservation with mercuric chloride (Liu et al., 2020; Wietz et al., 2021) and formalin (Stern et al., 2015) allows ribosomal metabarcoding of microbes in autonomously collected seawater. Also the nucleic acid stabilizers RNAlater and DNAgard can preserve environmental DNA (Gray et al., 2013; Rachel and Gieg, 2020), however requiring frozen storage in stabilizer solution or the concentration of microbial biomass on filters (Ottesen et al., 2011). Both reagents have been tested as preservative in automated microbial samplings (Boeuf et al., 2019; Formel et al., 2021; Poff et al., 2021), but can lead to DNA loss (Renshaw et al., 2015) and are likely unsuitable in remote regions where samples cannot be frozen immediately. Hence, although automated technologies – in particular comparative sampling across different regions – offer exciting perspectives, preservation method and storage time are challenging factors for microbial diversity studies (Sherr and Sherr, 1993; Rissanen et al., 2010; Metfies et al., 2017; Spens et al., 2017; Sano et al., 2020; Pratte and Kellogg, 2021).

In the present study, we examined how preservation and DNA extraction methods affect molecular microbial analyses after long-term storage of seawater samples. Specifically, we addressed DNA yields, PCR amplification efficiency and microbiome composition after sample storage for 10, 28, and 50 weeks (0°C) to mimic long-term autosampler deployments. The approach was chosen to match deployment conditions of autonomous samplers in polar waters, which are installed on moorings and typically serviced only once per year (e.g., von Appen et al., 2021). We evaluate the consistency of 16S and 18S rRNA sequence information obtained from samples after different periods of post-sampling storage. We focus on formalin and mercuric chloride as they are widely used preservatives (Supplementary Table S1) and functionally different, particularly with respect to long-term storage at *in situ* temperatures. Furthermore, we aimed to assess how results from freshly preserved samples align with those from legacy samples, and indeed allow decadal-scale characterization of ecosystem dynamics. Our results have implications for microbial time-series collected with automated samplers, both regarding short-term methodological aspects and long-term archiving of biodiversity information.

## Materials and methods

### Experimental design and sampling regime

Approx. 6 l of surface seawater were collected at the pier on Helgoland Island in the German Bight (54° 10′ 58.3″N, 7° 53′ 19.9″E) on March 30, 2017. The water sample was kept at 4°C in the dark for ~ 35 days, then well mixed and split into 40 ml subsamples. Five subsamples were directly filtered as reference. The following preservatives were added to four sets of five replicate subsamples: (i) saturated mercuric chloride (HgCl_2_) solution (0.15% *w*/*v* final concentration per sample), (ii) 20% formalin (1.8% *v*/*v* final concentration per sample), (iii) RNAlater (1% final concentration per sample), and (iv) DNAgard (1% final concentration per sample). Preserved 40 ml subsamples were stored in the dark at 0°C to mimic conditions during high-latitude mooring deployments. After 10, 28, and 50 weeks, respectively (hereafter referred to as 10w, 28w, 50w), five replicates per preservation method were subjected to DNA extraction with two different kits after filtering each 20 ml onto Isopore membrane filters (Millipore, Burlington, MA, United States; 0.2 μm pore size, 47 mm diameter). Filters were stored frozen at −20°C for the same amount of time until DNA extraction with the NucleoSpin II (NS; Macherey-Nagel, Germany) or PowerWater (PW; QIAGEN, Germany) kit following the manufacturers’ protocols. Filters from formalin-preserved samples were subjected to additional rinsing steps before DNA extraction following Bucklin and Allen (2004). DNA extracts were quantified using a Nanodrop 1000 photometer (Thermo Fisher Scientific, Germany) and stored frozen until library preparation.

### Amplicon sequencing

Libraries were prepared according to the standard instructions of the 16S Metagenomic Sequencing Library Preparation protocol (Illumina, San Diego, CA, United States). The V4 region of eukaryotic 18S rRNA genes was amplified using PCR primers 528F (5′-GCGGTAATTCCAGCTCCAA-3′; Elwood et al., 1985) and 964iR (5′-ACTTTCGTTCTTGATYRR-3′; Balzano et al., 2015). The V4-5 region of bacterial and archaeal 16S rRNA genes was amplified using primers 515F (5′-GTGYCAGCMGCCGCGGTAA-3′) and 926R (5′-CCGYCAATTYMTTTRAGTTT-3′; Parada et al., 2016). All PCRs had a final volume of 25 μl and contained 12.5 μl KAPA HiFi HotStart ReadyMix (Roche, Basel, Switzerland), 2.5 μl of each primer (1 μM) and 2.5 μl template. Amplification included initial denaturation (95°C, 3 min) followed by 25 cycles of denaturation (95°C, 30 s), annealing (55°C, 30 s), and extension (72°C, 30 s) with a single final extension (72°C, 5 min). 18S rRNA PCR products were gel-purified using the AMPure XP PCR purification kit (Beckman Coulter, Pasadena, CA, United States) according to the manufacturer’s protocol. All PCR products were quantified using a Quantus Fluorometer (Promega, Madison, WI, United States). Indices and sequencing adapters were attached *via* PCRs (final volume 50 μl), each containing 25 μl of KAPA HiFi HotStart ReadyMix (Roche), 5 μl of each Nextera XT Index Primer [1 μmol/l], 5 μl template (~5 ng DNA in total) and 10 μl PCR grade water. Amplification included initial denaturation (95°C, 3 min) followed by 8 cycles of denaturation (95°C, 30 s), annealing (55°C, 30 s), and extension (72°C, 30 s) with a single final extension (72°C, 5 min). 18S rRNA libraries were gel-purified using the AMPure XP PCR purification kit (Beckman Coulter). All libraries were quantified using a Quantus fluorometer (Promega) and sequenced using MiSeq and the MiSeq Reagent Kit V3 (2 × 300 bp) according to the manufacturer’s protocol (Illumina).

### Processing and analysis of amplicon reads

Reads were processed using Trimmomatic v0.38 (Bolger et al., 2014) by scanning each sequence from the 5′ to 3′ end, trimming the 3′ end if average Phred Q-score of < 8 in a sliding window of 3 bp. Paired ends were merged using VSEARCH v2.3.0 (Rognes et al., 2016), discarding pairs with <50 bp overlap and > 5 mismatches in the overlapping segment. To guarantee identical orientation, sequences were filtered so forward sequences occur before reverse complement sequences. If sequences did not match this pattern, their reverse complement was also scanned using cutadapt v1.17 (Martin, 2011), requiring minimum overlaps of 17 and 13 bp for forward and reverse primer sequences respectively, and only one mismatch. Primer sequences were truncated, and sequences feature-filtered using VSEARCH. Sequences were discarded if (i) < 300 bp or > 550 bp, (ii) containing ambiguous bases (assigned as RYSWKMBDHVN per IUPAC nomenclature), or (iii) having an expected error (sum of all base error probabilities) > 0.25. Each sample was independently dereplicated, and the abundance of each sequence added to the sequence header. Chimeras were sample-wise predicted *de novo* by VSEARCH with default settings and removed. Subsequently, only samples with at least 10,000 sequences were used. Cleaned sample files were pooled and dereplicated in total, keeping amplicon abundances in the sequence headers. The pooled file was used as input for OTU clustering with SWARM v2.2.2 (Mahé et al., 2014), using the most abundant amplicon of an OTU as representative for annotation. Sequences were annotated with the default classifier implemented in mothur v1.38.1 using the Protist Ribosomal database v4.11.1 (Guillou et al., 2013) and the Silva v132 database (Quast et al., 2013) for 18S and 16S rRNA amplicons respectively, with a confidence cut-off of 80. One representative sequence was used to annotate the full OTU cluster, discarding singletons as well as OTUs with < 0.005% relative abundance. Statistical evaluation was carried out with R v.4.1.1 in RStudio using packages phyloseq, ampvis2, iNEXT, vegan, ape, tidyverse and scico (McMurdie and Holmes, 2013; Oksanen et al., 2013; Hsieh et al., 2016; Andersen et al., 2018; Paradis and Schliep, 2019; Wickham et al., 2019; Crameri, 2021). As our 16S rRNA dataset contained almost no archaeal sequences, 16S results are only referred to as “bacteria”. Relative abundances were Hellinger-transformed (the square root of the relative abundance per OTU and sample), an ecologically relevant transformation to correct for the compositionality of amplicon sequence data (Legendre and Gallagher, 2001).

Preliminary sequence analyses showed that only HgCl_2_ and formalin performed well in our experimental design (Supplementary Figure S1). The nucleic acid stabilizers RNAlater and DNAgard were originally tested, since being used in some automated sampling approaches (Supplementary Table S1). However, as nucleic acid stabilizers are not designed for long-term sample storage without freezing, we omitted results from RNAlater and DNAgard from further analysis.

### Data and code availability

The entire workflow from raw sequence processing to statistical evaluation is available at https://github.com/matthiaswietz/MicroPreserve. Sequence data have been deposited in the European Nucleotide Archive (ENA) under accession number PRJEB43307, using the data brokerage service of the German Federation for Biological Data (GFBio) in compliance with MIxS standards (Yilmaz et al., 2011).

## Results and discussion

We evaluated microbial community composition in seawater samples following two different preservation methods, based on poisoning (HgCl_2_) and fixation by protein cross-links (formalin). The concentrations of HgCl_2_ and formalin, common preservatives to study water column biogeochemistry and microbiology, were at the higher end of the range typically used, aiming at the observation of the strongest preservative effect expected. HgCl_2_ (0.15% *w*/*v*) and formalin (1.8% *v*/*v*) concentrations correspond to those used in particle traps (Bauerfeind et al., 2009; Lampitt et al., 2010). HgCl_2_ concentrations in water sampler deployments can be tenfold lower (von Appen et al., 2021; Wietz et al., 2021) as biomass in seawater is commonly lower compared to particles.

### DNA yields and PCR amplification

Preservation with HgCl_2_ resulted in a higher proportion of successful DNA extractions compared to formalin (Table 1; Supplementary Figure S2) and approx. tenfold higher yields, despite pre-treatment of formalin-preserved samples (Bucklin and Allen, 2004). DNA-protein cross-linking through formalin may explain lower success rates and DNA yields. For both formalin and HgCl_2_, approximately twofold higher DNA yields were observed with PW extraction, likely corresponding to the combined bead-beating and enzymatic lysis compared to only chemical lysis with NS extraction (Yuan et al., 2015). In general, preservation decreased DNA yields two to fourfold compared to non-preserved controls. The impact of preservatives on DNA yields was observed at the earliest experimental time-point (10w), with no significant further decreases over the experimental period (Supplementary Figure S2). Hence, the chemical effect of preservatives is the major determinant of DNA yields, without further impact of prolonged storage, at least for up to 50 weeks. Independent of extraction kit, PCR amplification failures were ~ 50% for formalin compared to < 10% for HgCl_2_ (Table 1). Although formalin-preserved samples allowed DNA extraction and amplification in several cases, our results hence support that formalin can impede downstream molecular analyses (Hoffman et al., 2015; Reid et al., 2017).

**Table 1:** DNA yields and successful PCRs after preservation in comparison to the unpreserved reference, when extracted with either PowerWater (PW) or NucleoSpin (NS).

	Extractions with detectable DNA yield/sample number	DNA yield [ng μl^−1^]	Successful PCRs (16S/18S rRNA)
Reference; PowerWater	5/5	0.85 ± 0.2	5/5
Reference; NucleoSpin	5/5	0.2 ± 0.1	5/5
Mercuric chloride; PowerWater	15/15	0.24 ± 0.17	15/14
Mercuric chloride; NucleoSpin	13/15	0.03 ± 0.03	12/15
Formalin, PowerWater	5/15	0.2 ± 0.08	5/2
Formalin; NucleoSpin	11/15	0.01 ± 0.02	9/14

In some cases, PCR was successful despite NanoDrop did not detect DNA, probably related to detection sensitivity of the instrument. Reference: directly filtered environmental sample without preservation, immediately frozen at –20°C.

### Microbial community composition

**Figure 1:**
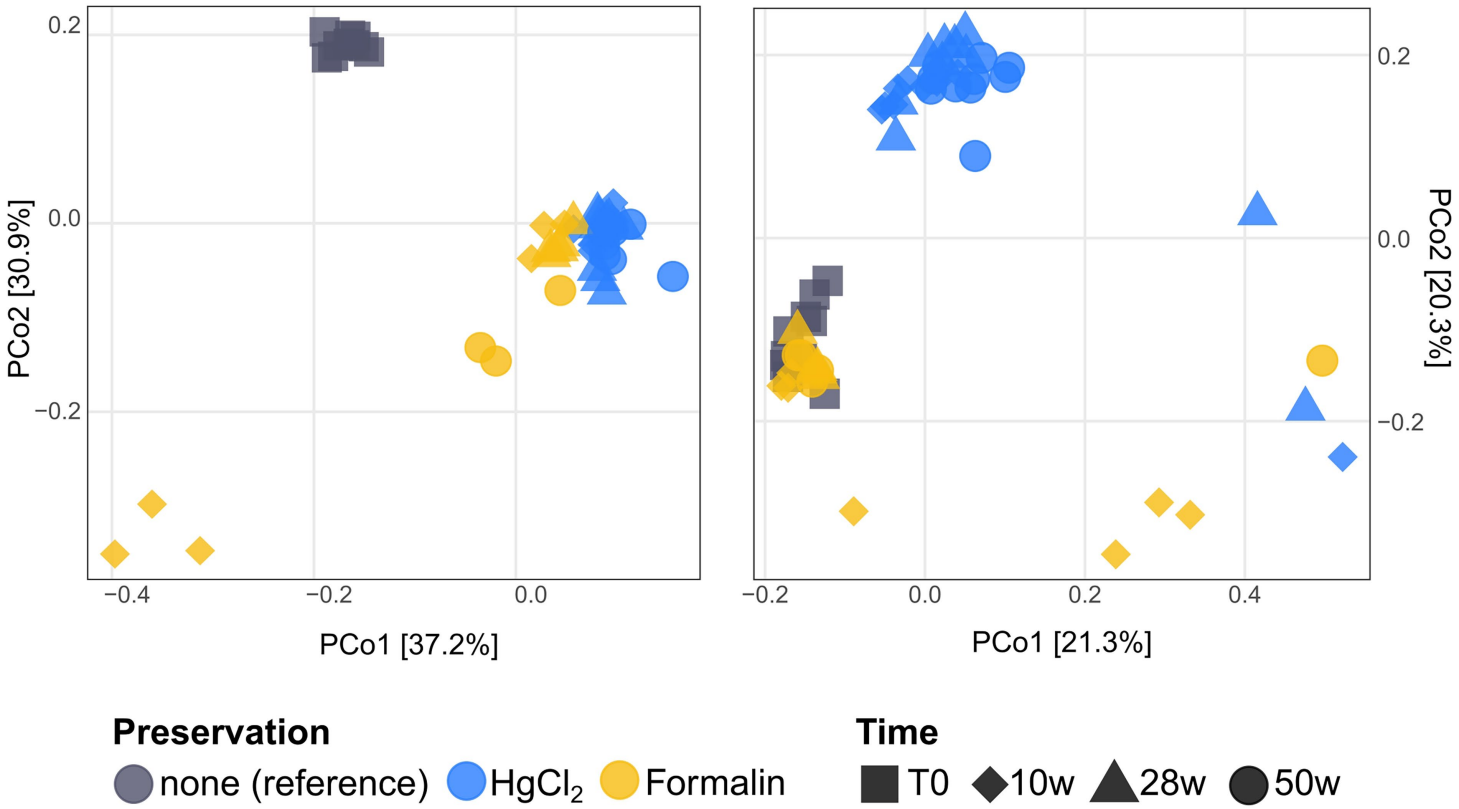
Principal Coordinates Analysis of Bray–Curtis dissimilarities of bacterial (left) and eukaryotic (right) communities after preservation and storage over different intervals. DNA extraction kits are not visually separated, as the influence of fixation significantly outweighs that of extraction.

We obtained a mean of 34,000 and 62,000 chimera-filtered 16S and 18S rRNA amplicon reads, respectively (Supplementary Table S2). Principal coordinates analysis revealed clear clustering of both eukaryotic and bacterial communities by preservation method (Figure 1; PERMANOVA, *p* < 0.01), with little effect of storage time or extraction kit. Hence, in line with DNA extraction and PCR results, preservation method is the major determinant of ribosomal metabarcoding results under the specific microbial community and storage conditions tested in this study.

#### Bacterial communities

**Figure 2:**
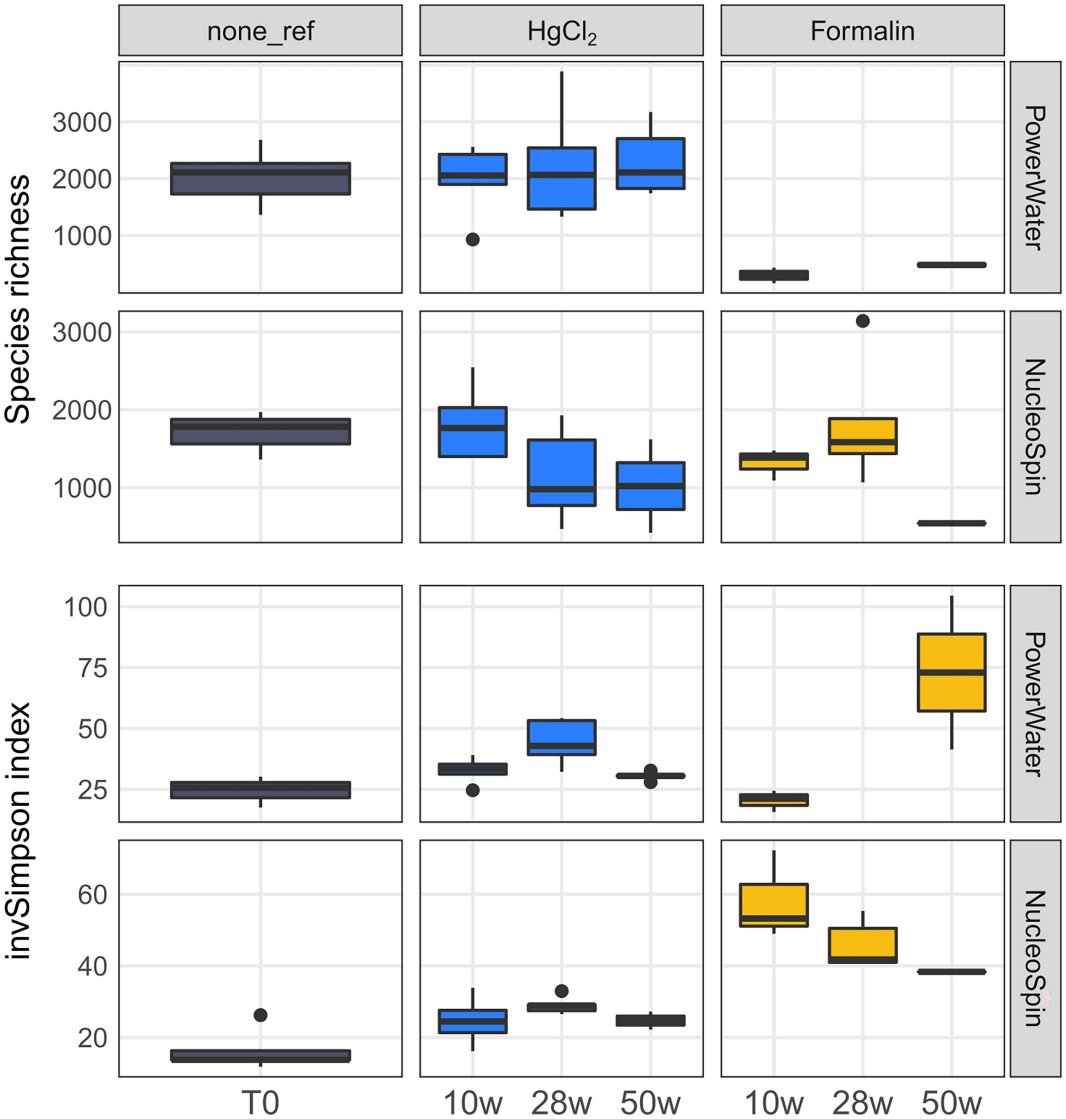
Bacterial species richness and inverse Simpson index by preservation, storage time, and DNA extraction. The number of samples per group is shown in Supplementary Table S2.

**Figure 3:**
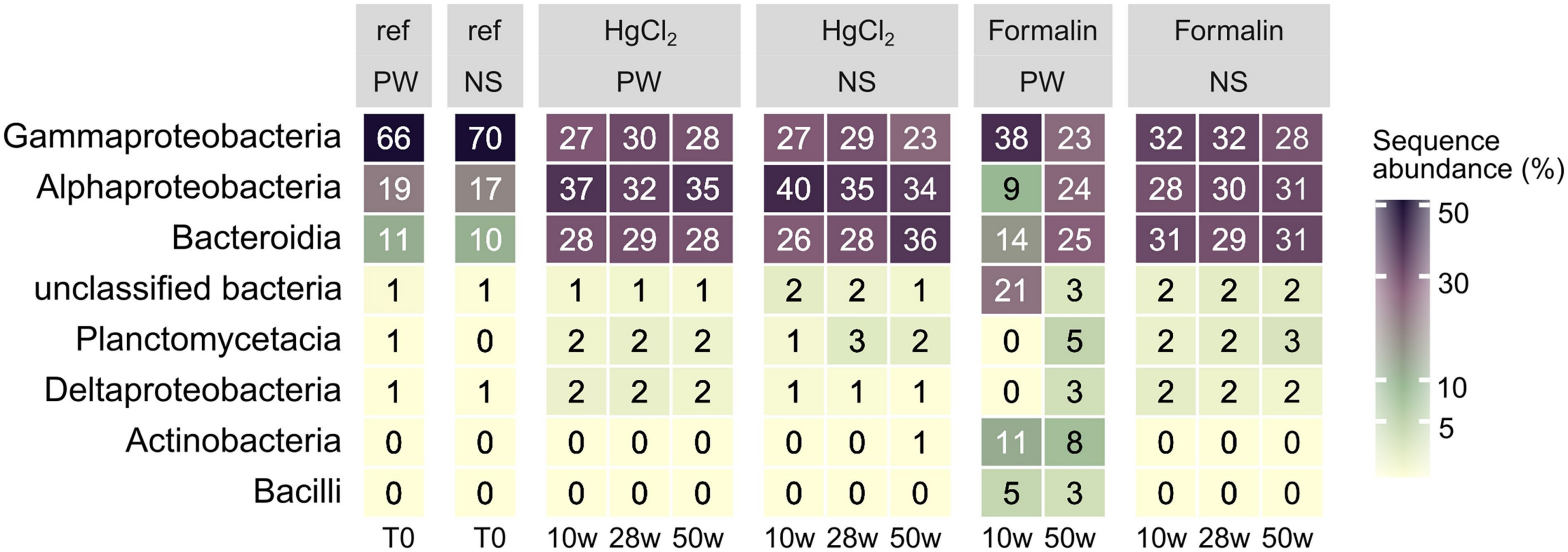
Relative abundances of major bacterial classes (average of all replicates per sampling event) by preservation, storage time, and DNA extraction. PW: PowerWater, NS: NucleoSpin.

**Figure 4:**
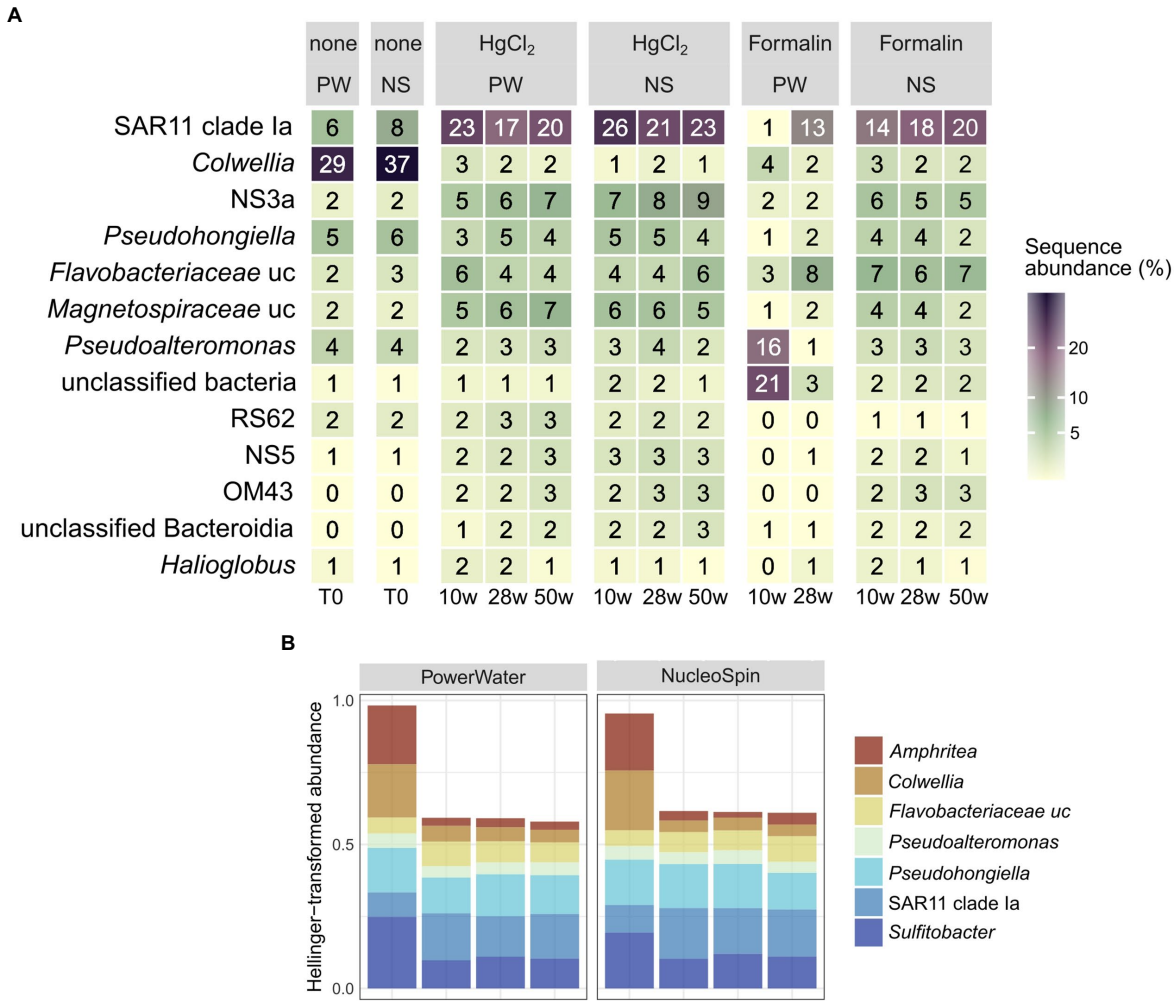
Relative abundances **(A)** and Hellinger-transformed relative abundances after HgCl_2_ preservation **(B)** of major bacterial genera (average of all replicates per sampling event) in relation to preservation, storage time, and DNA extraction. PW: PowerWater, NS: NucleoSpin, uc: unclassified.

Preservation significantly influenced bacterial community composition compared to the unpreserved reference (PERMANOVA, *p* < 0.001). However, differences to the unpreserved control were minor, with a taxonomic distance of ~ 0.2 particularly for HgCl_2_ samples and little change over time. In addition to preservation method, an effect of the extraction kit was observed. While HgCl_2_ + PW, HgCl_2_ + NS and formalin + NS performed comparably for bacterial communities, communities obtained from formalin + PW clustered separately (Supplementary Figure S3). The inverse Simpson index, considering both evenness and richness to determine alpha-diversity, was elevated after HgCl_2_ preservation (Figure 2; Kruskal–Wallis with Dunn’s *post hoc* test, *p* = 0.04). This concurred with higher relative abundances of planctomycetes, Deltaproteobacteria, and Actinobacteria (Figure 3), indicating that preservation can overestimate the rare biosphere. Among the major classes, preservation influenced the representation of alphaproteobacterial and gammaproteobacterial abundances (Figure 3), mainly relating to SAR11 clade Ia (higher) and *Colwellia* (lower abundances) respectively (Figure 4A). These taxa are at the lower and higher size spectrum of pelagic marine bacteria, respectively (Bowman, 2014; Giovannoni, 2017), indicating that preservation might favor smaller bacterial cells. Alternatively, cell wall structure and glycosylation (Dadon-Pilosof et al., 2017) might influence preservation efficiency. Compositionality effects can amplify such observations, but can be alleviated by normalizing relative abundances (Legendre and Gallagher, 2001; Weiss et al., 2017). Indeed, Hellinger-transformed relative abundances provided a more even picture of community structure (Figure 4B), with smaller differences for *Colwellia* while identifying highest variability *for Amphritea* (Gammaproteobacteria: Oceanospirillales). Previous studies have identified seasonal microbial dynamics in polar waters based on HgCl_2_ + PW preserved, autonomously collected samples (Liu et al., 2020; Wietz et al., 2021). Our results indicate that detection of *Colwellia* in such samples (Wietz et al., 2021) represented a true ecological finding, supported by stable OTU numbers from Alpha- and Gammaproteobacteria in HgCl_2_ + PW samples (Supplementary Figure S4).

#### Eukaryotic communities

**Figure 5:**
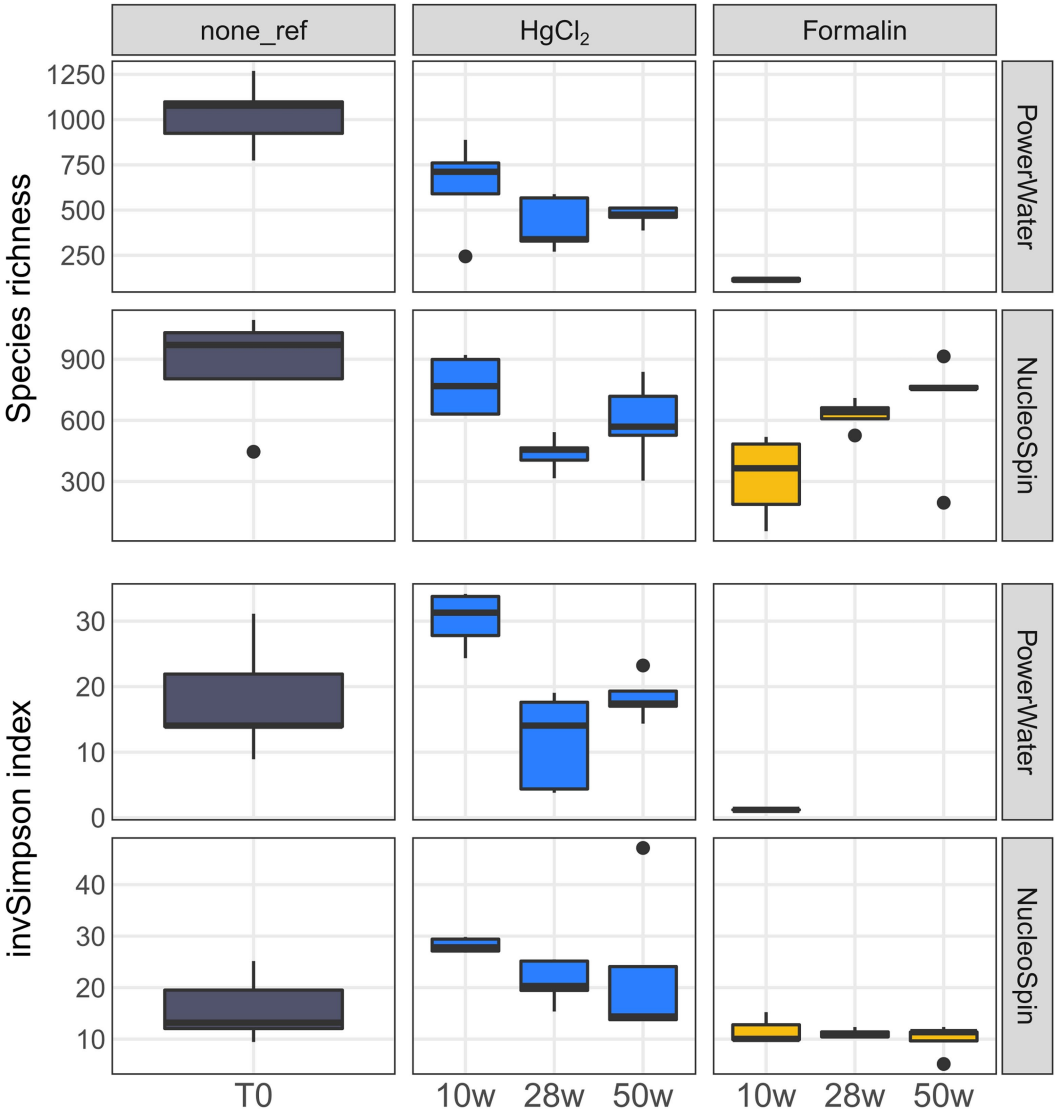
Eukaryotic species richness and inverse Simpson index by preservation, storage time, and DNA extraction. The number of samples per group is shown in Supplementary Table S2.

**Figure 6:**
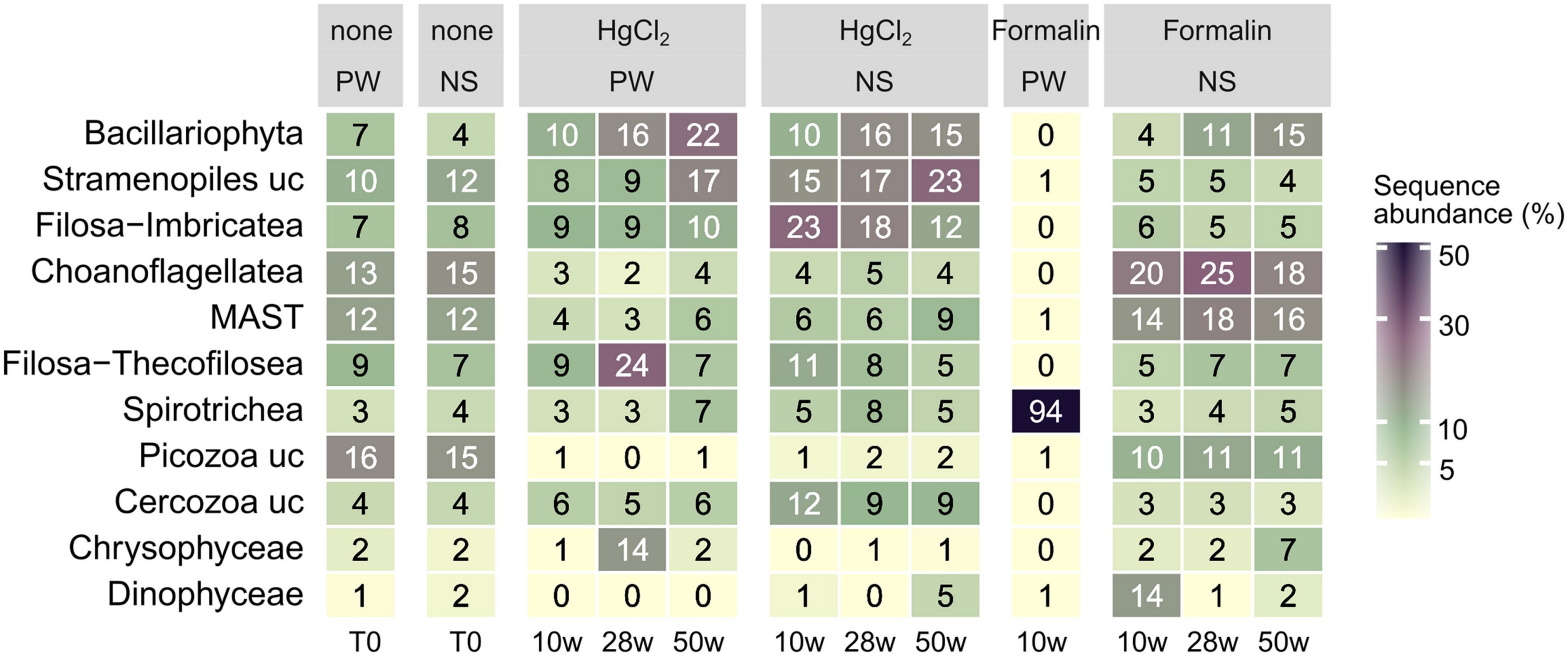
Relative abundances of major eukaryotic classes (average of all replicates per sampling event) by preservation, storage time, and DNA extraction. PW: PowerWater, NS: NucleoSpin, uc: unclassified.

**Figure 7:**
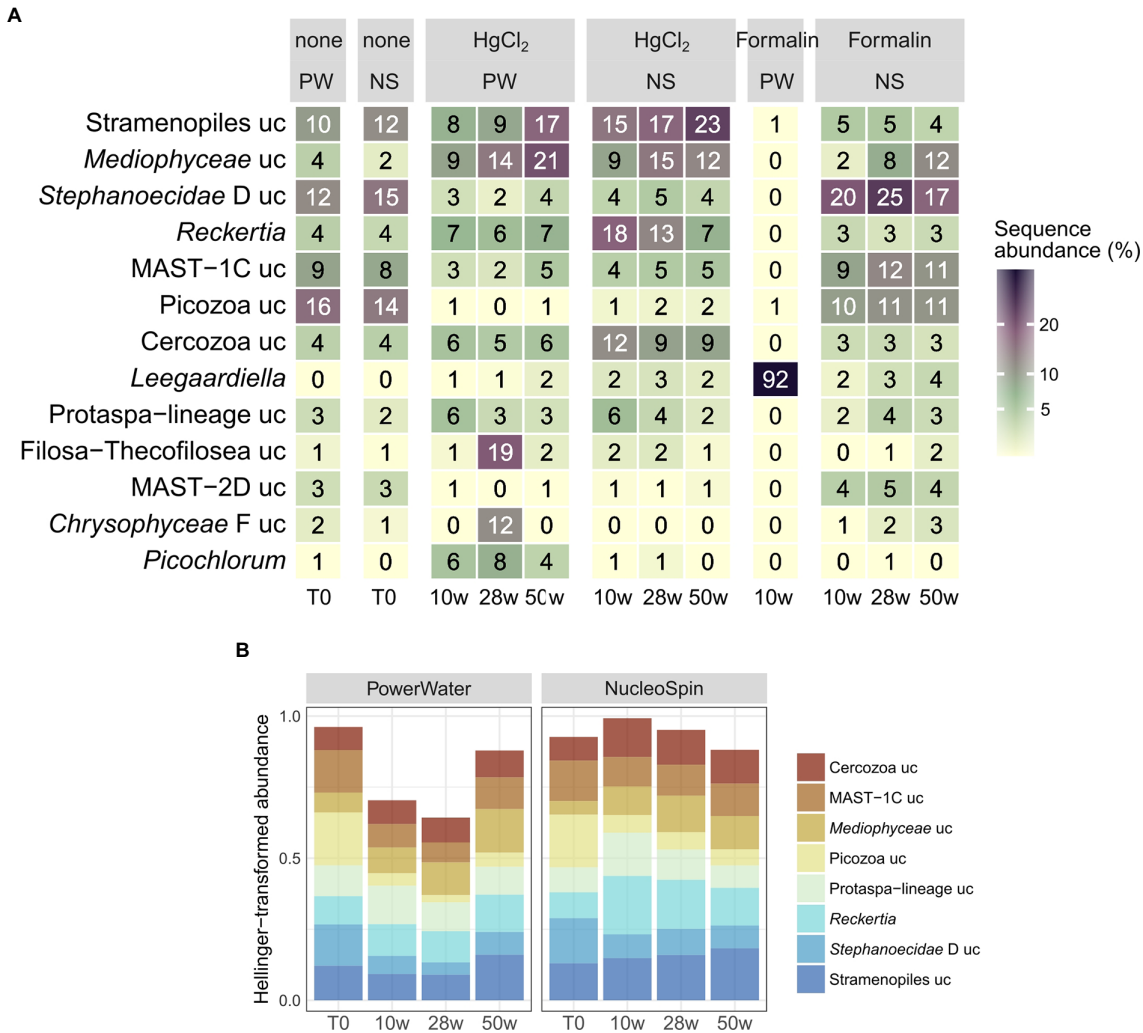
Relative abundances **(A)** and Hellinger-transformed relative abundances after HgCl_2_ preservation **(B)** of major eukaryotic genera (average of all replicates per sampling event) by preservation, storage time, and DNA extraction. PW: PowerWater, NS: NucleoSpin, uc: unclassified.

For eukaryotes, PW extraction of formalin-preserved samples largely failed (Table 1). Hence, we restricted assessment of method performance to formalin + NS and HgCl_2_ samples. Preservation significantly influenced eukaryotic community composition compared to the unpreserved reference (PERMANOVA, *p* < 0.001), albeit with minor differences to the unpreserved control (maximum taxonomic dissimilarities of ~0.3) comparable to bacteria. Formalin + NS, HgCl_2_ + NS and HgCl_2_ + PW performed similarly, providing comparable composition and diversity patterns compared to the reference (Figures 1, 5; Supplementary Figure S5). HgCl_2_ and formalin resulted in higher proportions of *Bacillariophyta* (i.e., diatoms) in comparison to the unpreserved reference. In addition, *Filosa-Imbricatea* and unclassified stramenopiles were overrepresented in HgCl_2_ + NS (Figure 6). The total number of OTUs detected within stramenopile groups was lower after both HgCl_2_ and formalin preservation (Supplementary Figure S4), contributing to an overall lower species richness compared to the reference (Figure 5). The relative abundances of picoplankton classes Picozoa, MAST and Choanoflagellata were most similar between the reference and formalin + NS (Figure 6). As opposed to the overrepresentation of smaller bacterial cells, HgCl_2_ preservation favored larger-size eukaryotes such as centric diatoms, with higher abundances of especially unclassified *Mediophyceae* compared to the reference (Figure 7A). If resources allow, additional microscopy (Metfies et al., 2017), quantitative PCR or flow cytometry are advised to assess the effect of preservation on cell numbers and/or size classes. As for bacteria, Hellinger-transformed data provided a more even picture of community structure (Figure 7B).

A comparison of results from the five technical replicates per treatment and time point allowed assessing the variability introduced by extraction and sequencing. Community structure in technical replicates were was highly reproducible for bacteria, but varied more for eukaryotes (Supplementary Figure S6). This observation potentially corresponds to disproportional distribution of large eukaryotic cells in some replicates, suggesting sample volumes should be maximized whenever possible. However, volumes and replicate numbers often need to be balanced with the desired temporal resolution, which can be challenging in remote locations relying on autonomous sampling.

## Conclusion

Understanding the ecological and biogeochemical roles of marine microbes substantially benefits from automated sampling in long-term ecological observatories. We herein assessed the combined effects of preservation, DNA extraction and storage time on ribosomal metabarcoding of bacterial and eukaryotic communities. These insights inform the design of automated microbial observation in remote waters, which rely on *in situ* preservation and *ex situ* extraction after extended storage between sample collection and retrieval of the sampler. We present four major conclusions:

1. HgCl_2_ + PW provided the best representation of bacterial diversity and composition, even after 1 year of storage. Despite altering some patterns observed in the original community, abundances of the major taxa were overall reproducible and differences restricted to only few taxa.
2. Formalin + NS performed best for eukaryotes, despite low DNA yields. Although logistically demanding in (automated) field studies, sampling volumes should be as large as possible to maximize the robustness of analyses. Hellinger or centered-log ratio transformations can counteract the inherent compositionality of amplicon data and provide a more reasonable picture of microbial dynamics.
3. For parallel assessment of bacteria and eukaryotes, we recommend HgCl_2_ + PW, as this provides good 16S and reasonable 18S rRNA sequence information from single DNA extracts. Our results indicate that the HgCl_2_ shortcomings in eukaryotes outweigh the formalin shortcomings in bacteria, indicating HgCl_2_ as most suitable for observatories aiming to study both groups based on DNA from the same samples. Nonetheless, individual time-series should perform similar benchmark studies, as the respective strengths and weaknesses might differ at other *in situ* temperatures and for other microbial communities.
4. In order to minimize bias, we recommend that the choice of preservation should also consider potentially desired comparisons with other sites, as well as other samples from the same observatory. For instance, in case of the FRAM observatory of the Alfred Wegener Institute, the use of HgCl_2_ + PW facilitates cross-comparability with metabarcoding of sinking particles from sediment traps, including decade-old legacy samples that are treated similarly.

## Data availability statement

The datasets presented in this study can be found in online repositories. The names of the repository/repositories and accession number(s) can be found at: https://www.ebi.ac.uk/ena, PRJEB43307.

## Author contributions

MW and KM analyzed the data and wrote the paper. CW performed the experimental work. CB, FJ, IS, and AB co-designed the study and contributed to writing. All authors contributed to the article and approved the submitted version.

## Funding

This work was supported by institutional funds of the Alfred Wegener Institute Helmholtz Centre for Polar and Marine Research, funds provided within the framework of the Helmholtz infrastructure program Frontiers in Arctic Marine Monitoring (FRAM), the Horizon 2020 project AtlantOS (grant ID 633211), and the ERC AdvGrant project ABYSS (grant ID 294757) to AB.

## Conflict of interest

The authors declare that the research was conducted in the absence of any commercial or financial relationships that could be construed as a potential conflict of interest.

## Publisher’s note

All claims expressed in this article are solely those of the authors and do not necessarily represent those of their affiliated organizations, or those of the publisher, the editors and the reviewers. Any product that may be evaluated in this article, or claim that may be made by its manufacturer, is not guaranteed or endorsed by the publisher.

## Supplementary material

SUPPLEMENTARY FIGURE S1 Principal Coordinates Analysis (PCoA) comparing community structure between all preservatives in relation to the unpreserved control, revealing marked separation of RNAlater and DNAgard samples despite similar read counts (Supplementary Table S2).

SUPPLEMENTARY FIGURE S2 DNA yields from reference (unpreserved, directly filtered) and preserved samples (HgCl_2_ and formalin) by extraction method. The number of samples per group is shown in Supplementary Table S2.

SUPPLEMENTARY FIGURE S3 Hierarchical clustering (complete linkage) of bacterial community composition based on Bray–Curtis dissimilarities (left) and Jaccard presence–absence (right). PW: PowerWater, NS: NucleoSpin.

SUPPLEMENTARY FIGURE S4 OTU numbers within the major bacterial **(A)** and eukaryotic **(B)** classes by preservation, storage time and extraction method.

SUPPLEMENTARY FIGURE S5 Hierarchical clustering (complete linkage) of eukaryotic community composition based on Bray-Curtis dissimilarities (left) and Jaccard presence–absence (right). PW: PowerWater, NS: NucleoSpin.

SUPPLEMENTARY FIGURE S6 Relative abundance heatmaps for bacteria **(A)** and eukaryotes **(B)** showing the abundance of major classes across all replicates by preservation, storage time and DNA extraction. Selected, markedly deviating replicates are encircled in red. Labels on the *x*-axis correspond to sample_titles of raw fastq files as deposited at the European Nucleotide Archive.

SUPPLEMENTARY TABLE S1 Selected marine microbial sampling efforts based on continuous, autonomous techniques.

SUPPLEMENTARY TABLE S2 Read counts and statistics from Swarm-OTU processing, and ENA accession numbers of original fastq files. Samples labeled “P-Buffer” were treated with phosphate buffer (i.e. unpreserved) and not considered in this study. Samples marked in red did not pass Swarm quality thresholds and were excluded from further analysis.

## References

[ref1] Andersen K. S. S. Kirkegaard R. H. Karst S. M. Albertsen M. (2018). ampvis2: an R package to analyse and visualise 16S rRNA amplicon data. *bioRxiv*. doi: 10.1101/299537 [Epub ahead of preprint].

[ref2] Bachy C. Sudek L. Choi C. J. Eckmann C. A. Nöthig E.-M. Metfies K.. (2022). Phytoplankton surveys in the Arctic Fram Strait demonstrate the tiny eukaryotic alga micromonas and other Picoprasinophytes contribute to deep sea export. Microorganisms 10:961. doi: 10.3390/microorganisms10050961, PMID: 35630405 PMC9144618

[ref3] Balzano S. Abs E. Leterme S. (2015). Protist diversity along a salinity gradient in a coastal lagoon. Aquat. Microb. Ecol. 74, 263–277. doi: 10.3354/ame01740

[ref4] Bauerfeind E. Nöthig E.-M. Beszczynska A. Fahl K. Kaleschke L. Kreker K.. (2009). Particle sedimentation patterns in the eastern Fram Strait during 2000–2005: results from the Arctic long-term observatory HAUSGARTEN. Deep-Sea Res. I Oceanogr. Res. Pap. 56, 1471–1487. doi: 10.1016/j.dsr.2009.04.011

[ref5] Boeuf D. Edwards B. R. Eppley J. M. Hu S. K. Poff K. E. Romano A. E.. (2019). Biological composition and microbial dynamics of sinking particulate organic matter at abyssal depths in the oligotrophic open ocean. Proc. Natl. Acad. Sci. 116, 11824–11832. doi: 10.1073/pnas.1903080116, PMID: 31127042 PMC6575173

[ref6] Bolger A. M. Lohse M. Usadel B. (2014). Trimmomatic: a flexible trimmer for Illumina sequence data. Bioinformatics 30, 2114–2120. doi: 10.1093/bioinformatics/btu170, PMID: 24695404 PMC4103590

[ref7] Bowman J. P. (2014). “The family *Colwelliaceae*” in The Prokaryotes: Gammaproteobacteria. eds. E. Rosenberg, E. F. DeLong, S. Lory, E. Stackenbrandt and F. Thompson (Berlin, Germany: Springer), 179–195.

[ref8] Bucklin A. Allen L. D. (2004). MtDNA sequencing from zooplankton after long-term preservation in buffered formalin. Mol. Phylogenet. Evol. 30, 879–882. doi: 10.1016/j.ympev.2003.11.002, PMID: 15012969

[ref9] Buttigieg P. L. Fadeev E. Bienhold C. Hehemann L. Offre P. Boetius A. (2018). Marine microbes in 4D—using time series observation to assess the dynamics of the ocean microbiome and its links to ocean health. Curr. Opin. Microbiol. 43, 169–185. doi: 10.1016/j.mib.2018.01.015, PMID: 29477022

[ref10] Crameri F. (2021). Scientific colour maps. doi: 10.5281/zenodo.5501399

[ref11] Dadon-Pilosof A. Conley K. R. Jacobi Y. Haber M. Lombard F. Sutherland K. R.. (2017). Surface properties of SAR11 bacteria facilitate grazing avoidance. Nat. Microbiol. 2, 1608–1615. doi: 10.1038/s41564-017-0030-5, PMID: 28970475

[ref12] Elwood H. J. Olsen G. J. Sogin M. L. (1985). The small-subunit ribosomal RNA gene sequences from the hypotrichous ciliates *Oxytricha nova* and *Stylonychia pustulata*. Mol. Biol. Evol. 2, 399–410. doi: 10.1093/oxfordjournals.molbev.a040362, PMID: 3939705

[ref13] Fischer G. Romero O. Merkel U. Donner B. Iversen M. Nowald N.. (2016). Deep ocean mass fluxes in the coastal upwelling off Mauritania from 1988 to 2012: variability on seasonal to decadal timescales. Biogeosciences 13, 3071–3090. doi: 10.5194/bg-13-3071-2016

[ref14] Formel N. Enochs I. C. Sinigalliano C. Anderson S. R. Thompson L. R. (2021). Subsurface automated samplers for eDNA (SASe) for biological monitoring and research. Hardware X 10:e00239. doi: 10.1016/j.ohx.2021.e00239, PMID: 35607674 PMC9123479

[ref15] Fuhrman J. A. Cram J. A. Needham D. M. (2015). Marine microbial community dynamics and their ecological interpretation. Nat. Rev. Microbiol. 13, 133–146. doi: 10.1038/nrmicro3417, PMID: 25659323

[ref16] Giovannoni S. J. (2017). SAR11 bacteria: The most abundant plankton in the oceans. Annu. Rev. Mar. Sci. 9, 231–255. doi: 10.1146/annurev-marine-010814-015934, PMID: 27687974

[ref17] Gray M. A. Pratte Z. A. Kellogg C. A. (2013). Comparison of DNA preservation methods for environmental bacterial community samples. FEMS Microbiol. Ecol. 83, 468–477. doi: 10.1111/1574-6941.12008, PMID: 22974342

[ref18] Guillou L. Bachar D. Audic S. Bass D. Berney C. Bittner L.. (2013). The Protist Ribosomal Reference database (PR2): a catalog of unicellular eukaryote small sub-unit rRNA sequences with curated taxonomy. Nucleic Acids Res. 41, D597–D604. doi: 10.1093/nar/gks1160, PMID: 23193267 PMC3531120

[ref19] Herfort L. Seaton C. Wilkin M. Roman B. Preston C. M. Marin R. III. (2016). Use of continuous, real-time observations and model simulations to achieve autonomous, adaptive sampling of microbial processes with a robotic sampler. Limnol. Oceanogr. Methods 14, 50–67. doi: 10.1002/lom3.10069

[ref20] Hoffman E. A. Frey B. L. Smith L. M. Auble D. T. (2015). Formaldehyde crosslinking: A tool for the study of chromatin complexes. J. Biol. Chem. 290, 26404–26411. doi: 10.1074/jbc.R115.651679, PMID: 26354429 PMC4646298

[ref21] Hsieh T. C. Ma K. H. Chao A. (2016). iNEXT: an R package for rarefaction and extrapolation of species diversity (Hill numbers). Methods Ecol. Evol. 7, 1451–1456. doi: 10.1111/2041-210X.12613

[ref22] Jørgensen B. B. Boetius A. (2007). Feast and famine — microbial life in the deep-sea bed. Nat. Rev. Microbiol. 5, 770–781. doi: 10.1038/nrmicro1745, PMID: 17828281

[ref23] Lampitt R. S. Salter I. de Cuevas B. A. Hartman S. Larkin K. E. Pebody C. A. (2010). Long-term variability of downward particle flux in the deep Northeast Atlantic: causes and trends. Deep-Sea Res. II Top. Stud. Oceanogr. 57, 1346–1361. doi: 10.1016/j.dsr2.2010.01.011

[ref24] Lee C. Hegdes J. Wakeham S. Zhu N. (1992). Effectiveness of various treatments in retarding microbial activity in sediment trap material and their effects on the collection of swimmers. Limnol. Oceanogr. 37, 117–130. doi: 10.4319/lo.1992.37.1.0117

[ref25] Legendre P. Gallagher E. D. (2001). Ecologically meaningful transformations for ordination of species data. Oecologia 129, 271–280. doi: 10.1007/s004420100716, PMID: 28547606

[ref26] Lindsay D. J. (2021). Stealthy tracking of deep ocean organisms with Mesobot. Sci. Robot. 6:eabj3949. doi: 10.1126/scirobotics.abj3949, PMID: 34135119

[ref27] Liu Y. Blain S. Crispi O. Rembauville M. Obernosterer I. (2020). Seasonal dynamics of prokaryotes and their associations with diatoms in the Southern Ocean as revealed by an autonomous sampler. Environ. Microbiol. 22, 3968–3984. doi: 10.1111/1462-2920.15184, PMID: 32755055

[ref28] Mahé F. Rognes T. Quince C. de Vargas C. Dunthorn M. (2014). Swarm: robust and fast clustering method for amplicon-based studies. Peer J. 2:e593. doi: 10.7717/peerj.593, PMID: 25276506 PMC4178461

[ref29] Martin M. (2011). Cutadapt removes adapter sequences from high-throughput sequencing reads. EMBnet.journal 17, 10–12. doi: 10.14806/ej.17.1.200

[ref30] McMurdie P. J. Holmes S. (2013). Phyloseq: an R package for reproducible interactive analysis and graphics of microbiome census data. PLoS One 8:e61217. doi: 10.1371/journal.pone.0061217, PMID: 23630581 PMC3632530

[ref31] Metfies K. Bauerfeind E. Wolf C. Sprong P. Frickenhaus S. Kaleschke L.. (2017). Protist communities in moored long-term sediment traps (Fram Strait, Arctic)–preservation with mercury chloride allows for PCR-based molecular genetic analyses. Front. Mar. Sci. 4:301. doi: 10.3389/fmars.2017.00301

[ref32] Moore S. K. Mickett J. B. Doucette G. J. Adams N. G. Mikulski C. M. Birch J. M.. (2021). An autonomous platform for near real-time surveillance of harmful algae and their toxins in dynamic coastal shelf environments. J. Mar. Sci. Eng. 9:336. doi: 10.3390/jmse9030336

[ref33] Nöthig E.-M. Lalande C. Fahl K. Metfies K. Salter I. Bauerfeind E. (2020). Annual cycle of downward particle fluxes on each side of the Gakkel ridge in the Central Arctic Ocean. Philos. Trans. R. Soc. A Math. Phys. Eng. Sci. 378:20190368. doi: 10.1098/rsta.2019.0368, PMID: 32862819 PMC7481669

[ref34] Oksanen J. Blanchet F. G. Kindt R. Legendre P. Minchin P. R. O’Hara R. B.. (2013). Vegan: community ecology package.

[ref400] Ottesen E. A. Mari R. A. Preston C. M. Young C. R. Ryan J. P. Scholin C. A.. (2011). Metatranscriptomic analysis of autonomously collected and preserved marine bacterioplankton. ISME J. 5, 1881–1895. doi: 10.1038/ismej.2011.70, PMID: 21716310 PMC3223310

[ref35] Parada A. E. Needham D. M. Fuhrman J. A. (2016). Every base matters: assessing small subunit rRNA primers for marine microbiomes with mock communities, time series and global field samples. Environ. Microbiol. 18, 1403–1414. doi: 10.1111/1462-2920.13023, PMID: 26271760

[ref36] Paradis E. Schliep K. (2019). Ape 5.0: an environment for modern phylogenetics and evolutionary analyses in R. Bioinformatics 35, 526–528. doi: 10.1093/bioinformatics/bty633, PMID: 30016406

[ref37] Poff K. E. Leu A. O. Eppley J. M. Karl D. M. DeLong E. F. (2021). Microbial dynamics of elevated carbon flux in the open ocean’s abyss. Proc. Natl. Acad. Sci. U. S. A. 118:e2018269118. doi: 10.1073/pnas.2018269118, PMID: 33479184 PMC7848738

[ref38] Pratte Z. A. Kellogg C. A. (2021). Comparison of preservation and extraction methods on five taxonomically disparate coral microbiomes. Front. Mar. Sci. 8:684161. doi: 10.3389/fmars.2021.684161

[ref39] Quast C. Pruesse E. Yilmaz P. Gerken J. Schweer T. Yarza P.. (2013). The SILVA ribosomal RNA gene database project: improved data processing and web-based tools. Nucleic Acids Res. 41, D590–D596. doi: 10.1093/nar/gks1219, PMID: 23193283 PMC3531112

[ref40] Rachel N. M. Gieg L. M. (2020). Preserving microbial community integrity in oilfield produced water. Front. Microbiol. 11:581387. doi: 10.3389/fmicb.2020.581387 33193212 PMC7604316

[ref41] Ramondenc S. Nöthig E.-M. Hufnagel L. Bauerfeind E. Busch K. Knüppel N.. (2022). Effects of Atlantification and changing sea-ice dynamics on zooplankton community structure and carbon flux between 2000 and 2016 in the eastern Fram Strait. Limnol. Oceanogr. doi: 10.1002/lno.12192 (in press).

[ref42] Reid K. M. Maistry S. Ramesar R. Heathfield L. J. (2017). A review of the optimisation of the use of formalin fixed paraffin embedded tissue for molecular analysis in a forensic post-mortem setting. Forensic Sci. Int. 280, 181–187. doi: 10.1016/j.forsciint.2017.09.020, PMID: 29078160

[ref43] Renshaw M. A. Olds B. P. Jerde C. L. McVeigh M. M. Lodge D. M. (2015). The room temperature preservation of filtered environmental DNA samples and assimilation into a phenol–chloroform–isoamyl alcohol DNA extraction. Mol. Ecol. Resour. 15, 168–176. doi: 10.1111/1755-0998.12281, PMID: 24834966 PMC4312482

[ref44] Rissanen A. J. Kurhela E. Aho T. Oittinen T. Tiirola M. (2010). Storage of environmental samples for guaranteeing nucleic acid yields for molecular microbiological studies. Appl. Microbiol. Biotechnol. 88, 977–984. doi: 10.1007/s00253-010-2838-2, PMID: 20730531

[ref45] Rognes T. Flouri T. Nichols B. Quince C. Mahé F. (2016). VSEARCH: a versatile open source tool for metagenomics. Peer J. 4:e2584. doi: 10.7717/peerj.2584, PMID: 27781170 PMC5075697

[ref46] Salter I. Kemp A. E. S. Moore C. M. Lampitt R. S. Wolff G. A. Holtvoeth J. (2012). Diatom resting spore ecology drives enhanced carbon export from a naturally iron-fertilized bloom in the Southern Ocean. Glob. Biogeochem. Cycles 26:GB1014. doi: 10.1029/2010GB003977

[ref47] Salter I. Lampitt R. Sanders R. Poulton A. Kemp A. Boorman B.. (2007). Estimating carbon, silica and diatom export from a naturally fertilised phytoplankton bloom in the Southern Ocean using PELAGRA: A novel drifting sediment trap. Deep-Sea Res. I-Top. Stud. Oceanogr. 54, 2233–2259. doi: 10.1016/j.dsr2.2007.06.008

[ref48] Salter I. Schiebel R. Ziveri P. Movellan A. Lampitt R. Wolff G. A. (2014). Carbonate counter pump stimulated by natural iron fertilization in the polar frontal zone. Nat. Geosci. 7, 885–889. doi: 10.1038/ngeo2285

[ref49] Sano M. Makabe R. Kurosawa N. Moteki M. Odate T. (2020). Effects of Lugol’s iodine on long-term preservation of marine plankton samples for molecular and stable carbon and nitrogen isotope analyses. Limnol. Oceanogr. Methods 18, 635–643. doi: 10.1002/lom3.10390

[ref50] Sherr E. B. Sherr B. F. (1993). “Preservation and Storage of Samples for Enumeration of Heterotrophic Protists,” in Handbook of Methods in Aquatic Microbial Ecology. eds. P. F. Kemp, B. F. Sherr, E. B. Sherr and J. J. Cole (Boca Raton, FL: CRC Press), 207–212.

[ref51] Spens J. Evans A. R. Halfmaerten D. Knudsen S. W. Sengupta M. E. Mak S. S. T.. (2017). Comparison of capture and storage methods for aqueous microbial eDNA using an optimized extraction protocol: advantage of enclosed filter. Methods Ecol. Evol. 8, 635–645. doi: 10.1111/2041-210X.12683

[ref52] Stern R. F. Picard K. T. Hamilton K. M. Walne A. Tarran G. A. Mills D.. (2015). Novel lineage patterns from an automated water sampler to probe marine microbial biodiversity with ships of opportunity. Prog. Oceanogr. 137, 409–420. doi: 10.1016/j.pocean.2015.04.015

[ref53] Sunagawa S. Coelho L. P. Chaffron S. Kultima J. R. Labadie K. Salazar G.. (2015). Structure and function of the global ocean microbiome. Science 348:1261359. doi: 10.1126/science.1261359 25999513

[ref54] Truelove N. K. Patin N. V. Min M. Pitz K. J. Preston C. M. Yamahara K. M.. (2022). Expanding the temporal and spatial scales of environmental DNA research with autonomous sampling. Environ. DNA 4, 972–984. doi: 10.1002/edn3.299

[ref55] UNESCO-IOC (1994). Protocols for the Joint Global Ocean Flux Study (JGOFS) Core Measurements. Paris: UNESCO.

[ref56] Valencia B. Stukel M. R. Allen A. E. McCrow J. P. Rabines A. Landry M. R. (2022). Microbial communities associated with sinking particles across an environmental gradient from coastal upwelling to the oligotrophic ocean. Deep-Sea Res. I Oceanogr. Res. Pap. 179:103668. doi: 10.1016/j.dsr.2021.103668

[ref57] von Appen W.-J. Waite A. M. Bergmann M. Bienhold C. Boebel O. Bracher A.. (2021). Sea-ice derived meltwater stratification slows the biological carbon pump: results from continuous observations. Nat. Commun. 12:7309. doi: 10.1038/s41467-021-26943-z, PMID: 34911949 PMC8674288

[ref58] Weiss S. Xu Z. Z. Peddada S. Amir A. Bittinger K. Gonzalez A.. (2017). Normalization and microbial differential abundance strategies depend upon data characteristics. Microbiome 5:27. doi: 10.1186/s40168-017-0237-y, PMID: 28253908 PMC5335496

[ref59] Wickham H. Averick M. Bryan J. Chang W. McGowan L. D. François R.. (2019). Welcome to the Tidyverse. J. Open Source Softw. 4:1686. doi: 10.21105/joss.01686

[ref60] Wietz M. Bienhold C. Metfies K. Torres-Valdés S. von Appen W.-J. Salter I.. (2021). The polar night shift: seasonal dynamics and drivers of Arctic Ocean microbiomes revealed by autonomous sampling. ISME Commun. 1:76. doi: 10.1038/s43705-021-00074-4 PMC9723606 37938651

[ref61] Yamahara K. M. Preston C. M. Birch J. Walz K. Marin R. Jensen S.. (2019). *In situ* autonomous acquisition and preservation of marine environmental DNA using an autonomous underwater vehicle. Front. Mar. Sci. 6:373. doi: 10.3389/fmars.2019.00373

[ref62] Yilmaz P. Kottmann R. Field D. Knight R. Cole J. R. Amaral-Zettler L.. (2011). Minimum information about a marker gene sequence (MIMARKS) and minimum information about any (x) sequence (MIxS) specifications. Nat. Biotechnol. 29, 415–420. doi: 10.1038/nbt.1823, PMID: 21552244 PMC3367316

[ref63] Yuan J. Li M. Lin S. (2015). An improved DNA extraction method for efficient and quantitative recovery of phytoplankton diversity in natural assemblages. PLoS One 10:e0133060. doi: 10.1371/journal.pone.0133060, PMID: 26218575 PMC4517865

[ref64] Zhang Y. Ryan J. P. Hobson B. W. Kieft B. Romano A. Barone B.. (2021). A system of coordinated autonomous robots for Lagrangian studies of microbes in the oceanic deep chlorophyll maximum. Sci. Robot. 6:eabb9138. doi: 10.1126/scirobotics.abb9138, PMID: 34043577

[ref65] Zhang Y. Ryan J. P. Kieft B. Hobson B. W. McEwen R. S. Godin M. A.. (2019). Targeted sampling by autonomous underwater vehicles. Front. Mar. Sci. 6:415. doi: 10.3389/fmars.2019.00415

[ref66] Zúñiga D. Sanchez-Vidal A. Flexas M. M. Carroll D. Rufino M. M. Spreen G.. (2021). Sinking diatom assemblages as a key driver for deep carbon and silicon export in the Scotia Sea (Southern Ocean). Front. Earth Sci. 9:579198. doi: 10.3389/feart.2021.579198

