# Supplementary figures and images for: Impact of preservation method and storage period on ribosomal metabarcoding of marine microbes: Implications for remote automated samplings
Source: Front Microbiol. 2022 Sep 7;13:999925. doi: 10.3389/fmicb.2022.999925 (PMC9490091; doi:10.3389/fmicb.2022.999925)

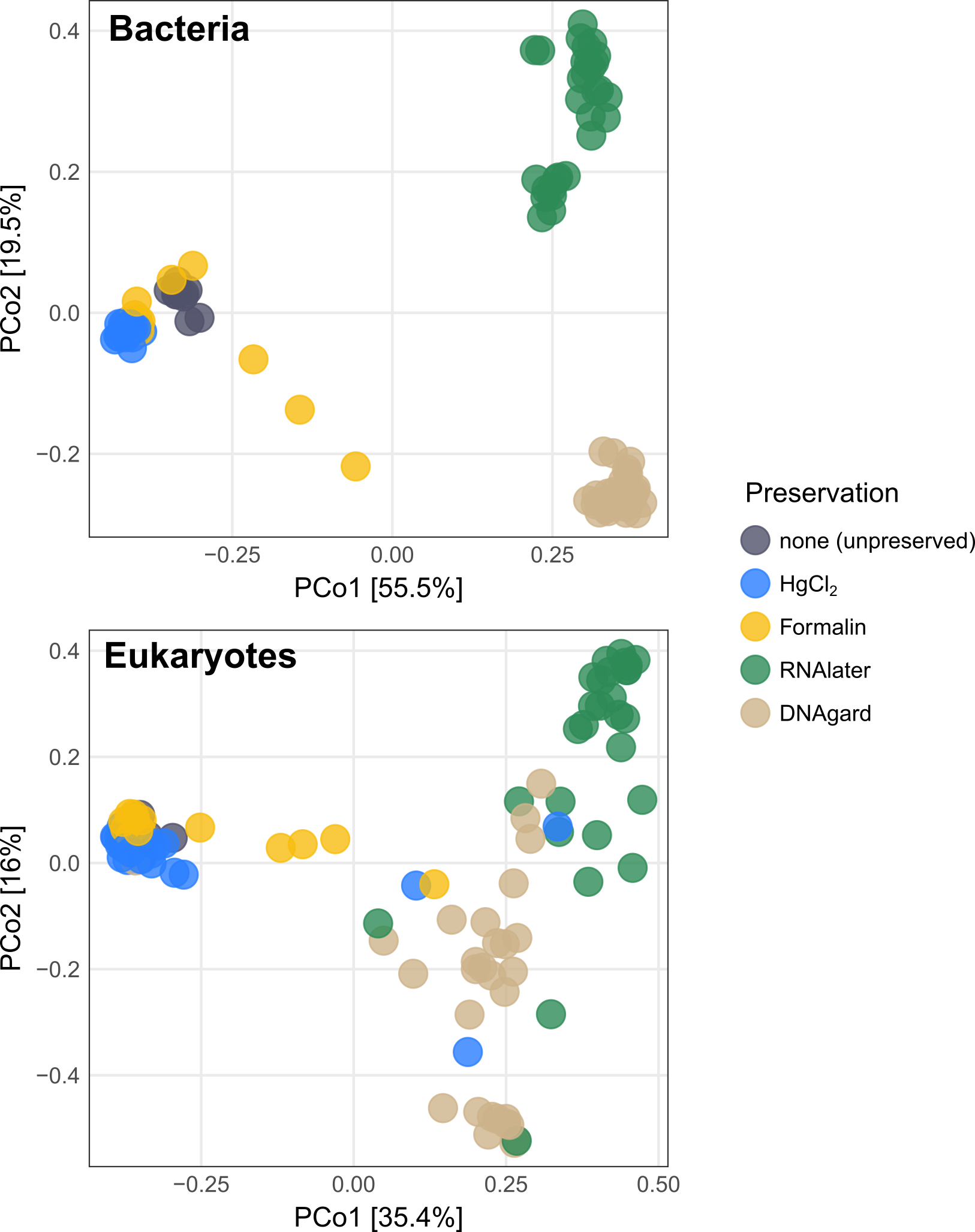

Supplement: SUPPLEMENTARY FIGURE S1 — Principal Coordinates Analysis (PCoA) comparing community structure between all preservatives in relation to the unpreserved control, revealing marked separation of RNAlater and DNAgard samples despite similar read counts (Supplementary Table S2). [file Image_1.PNG]

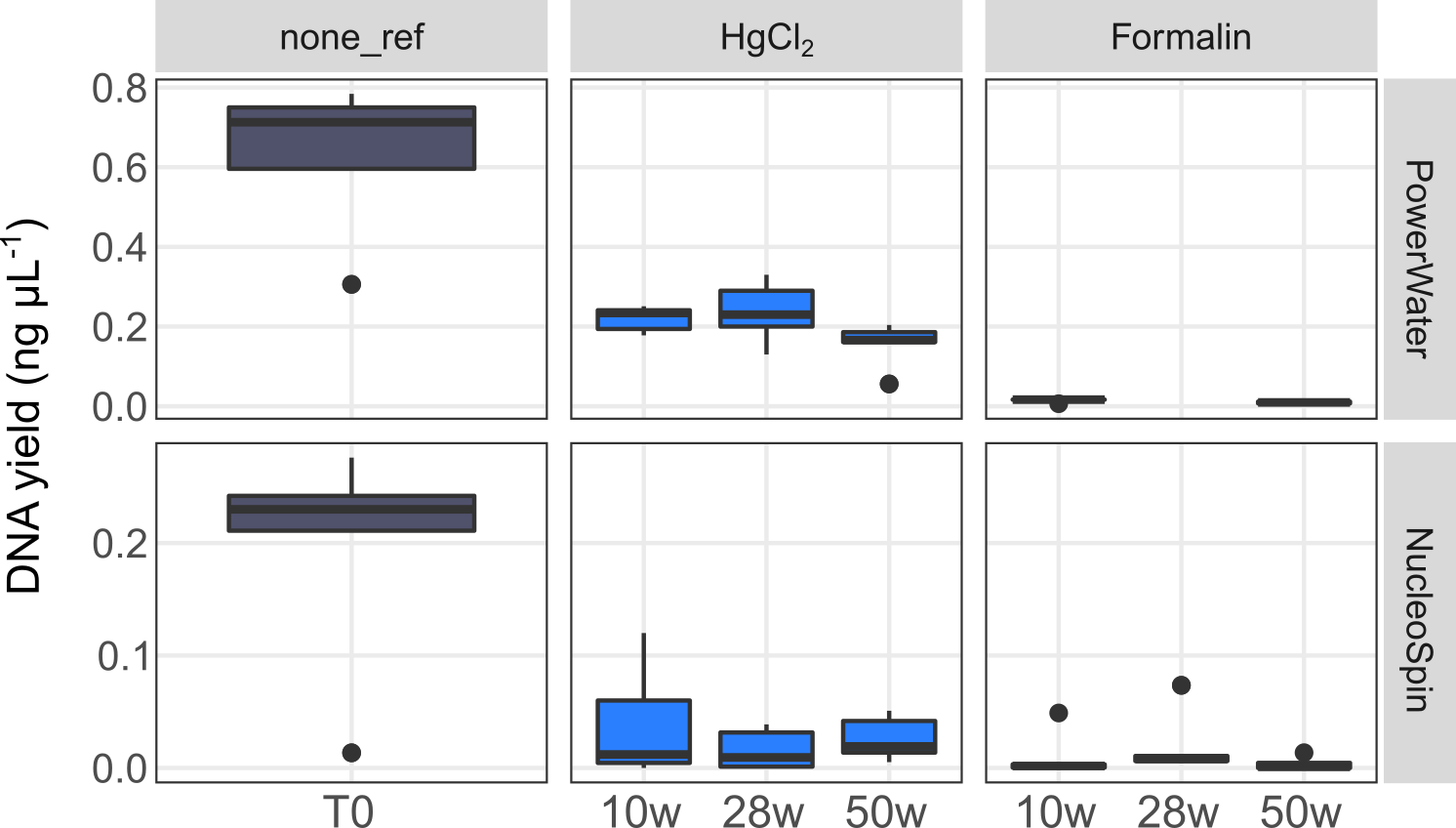

Supplement: SUPPLEMENTARY FIGURE S2 — DNA yields from reference (unpreserved, directly filtered) and preserved samples (HgCl2 and formalin) by extraction method. The number of samples per group is shown in Supplementary Table S2. [file Image_2.PNG]

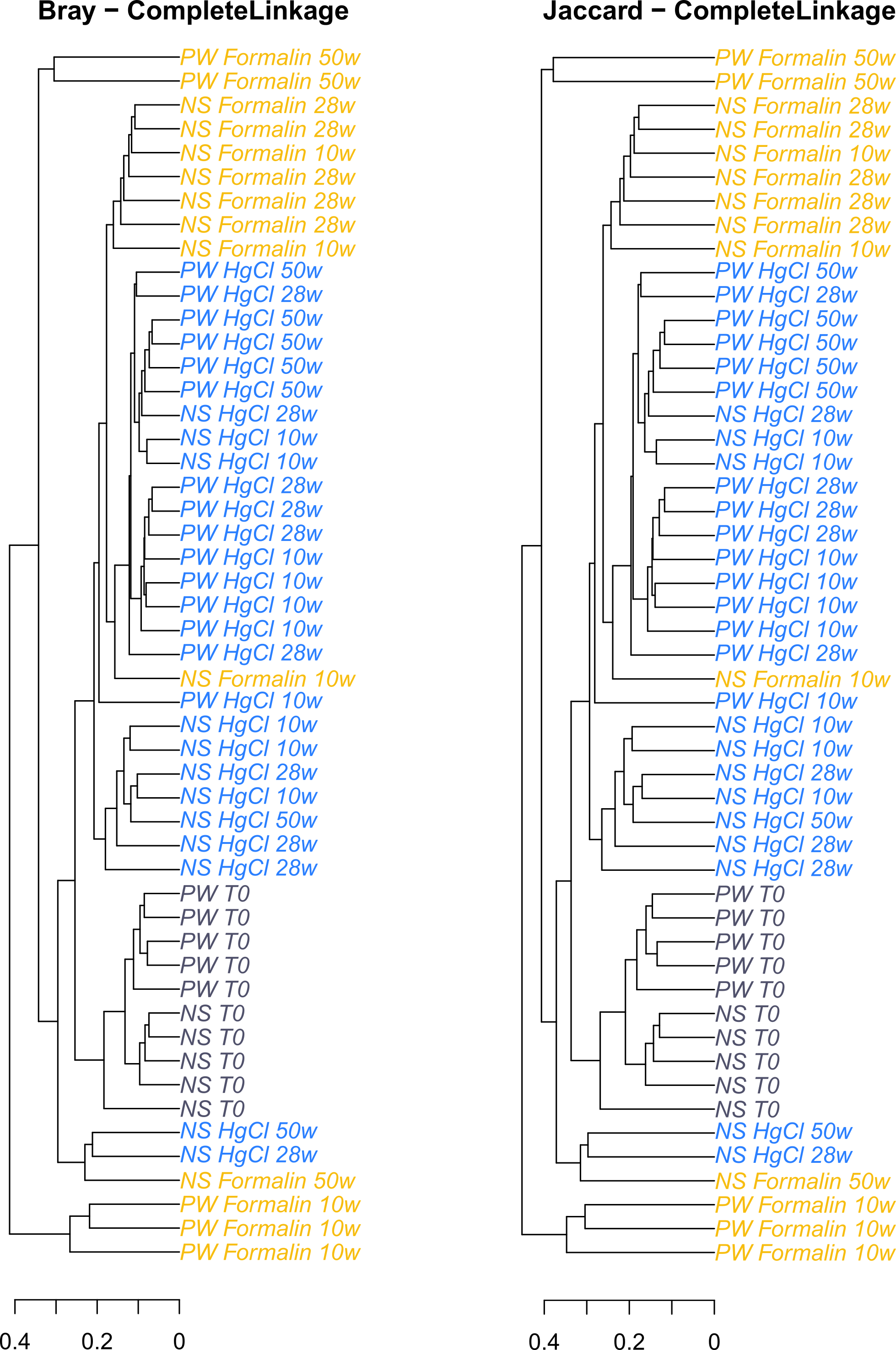

Supplement: SUPPLEMENTARY FIGURE S3 — Hierarchical clustering (complete linkage) of bacterial community composition based on Bray–Curtis dissimilarities (left) and Jaccard presence–absence (right). PW: PowerWater, NS: NucleoSpin. [file Image_3.PNG]

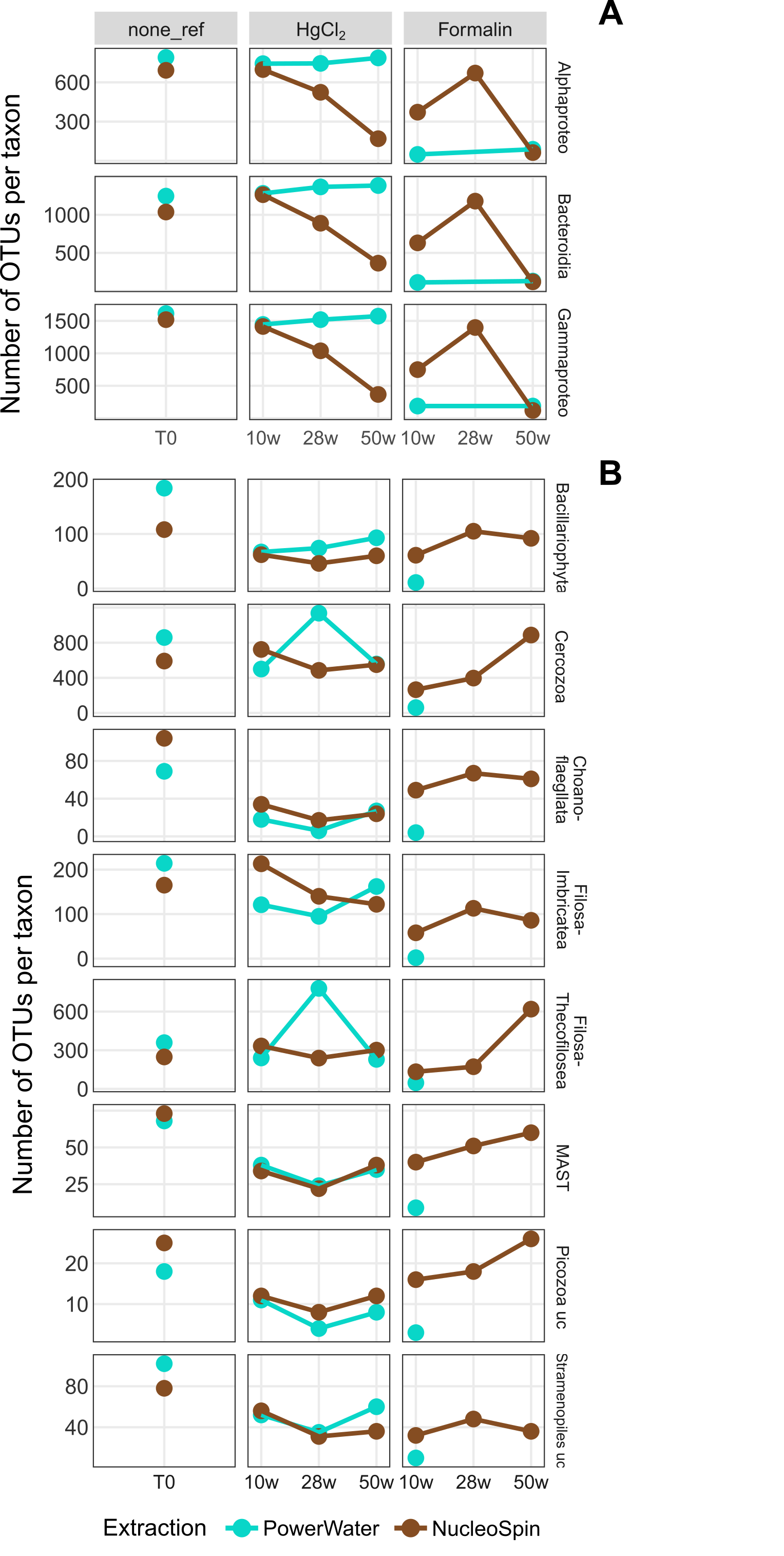

Supplement: SUPPLEMENTARY FIGURE S4 — OTU numbers within the major bacterial (A) and eukaryotic (B) classes by preservation, storage time and extraction method. [file Image_4.PNG]

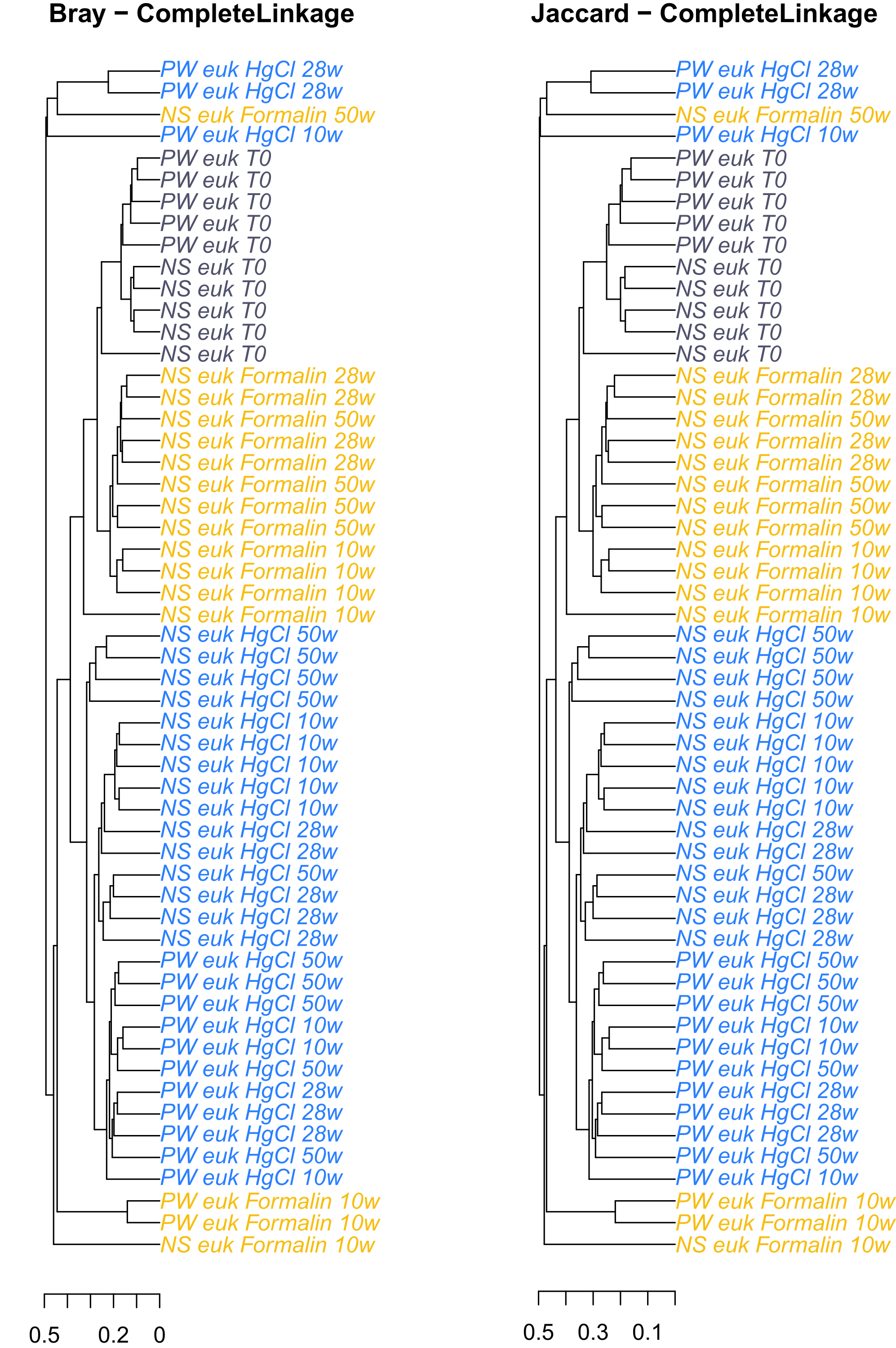

Supplement: SUPPLEMENTARY FIGURE S5 — Hierarchical clustering (complete linkage) of eukaryotic community composition based on Bray-Curtis dissimilarities (left) and Jaccard presence–absence (right). PW: PowerWater, NS: NucleoSpin. [file Image_5.PNG]

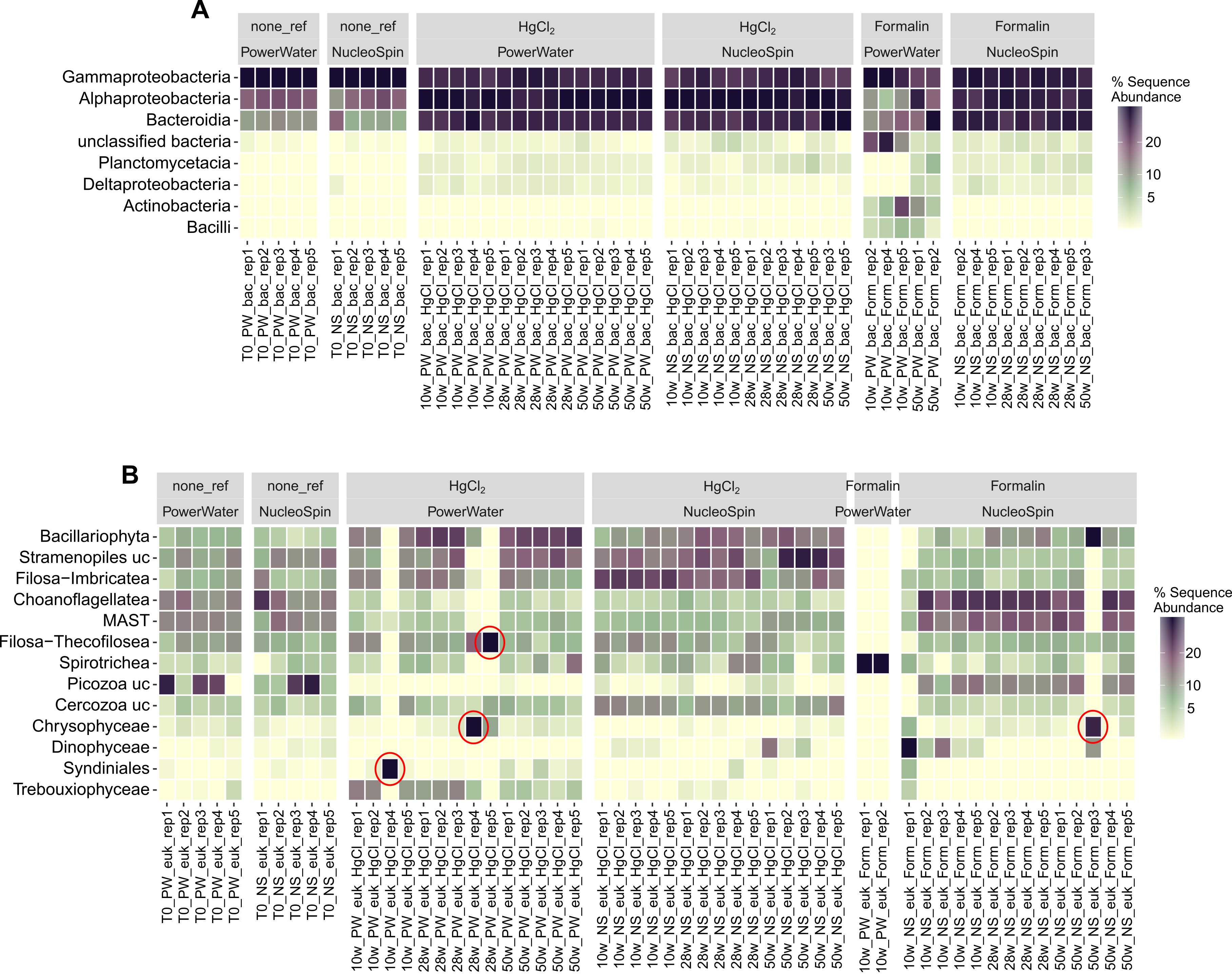

Supplement: SUPPLEMENTARY FIGURE S6 — Relative abundance heatmaps for bacteria (A) and eukaryotes (B) showing the abundance of major classes across all replicates by preservation, storage time and DNA extraction. Selected, markedly deviating replicates are encircled in red. Labels on the x-axis correspond to sample_titles of raw fastq files as deposited at the European Nucleotide Archive. [file Image_6.PNG]
